# Dengue-Related Hemophagocytic Lymphohistiocytosis in an Adult: A Case Report and Brief Update

**DOI:** 10.1055/s-0044-1788687

**Published:** 2024-09-25

**Authors:** Anju Dinkar, Jitendra Singh, Nilesh Kumar, Kailash Kumar, Santosh Kumar Singh

**Affiliations:** 1 Department of Microbiology, Sanjay Gandhi Postgraduate Institute of Medical Sciences, Lucknow, Uttar Pradesh, India; 2 Department of Microbiology, Institute of Medical Sciences, Banaras Hindu University, Varanasi, Uttar Pradesh, India; 3 Department of General Medicine, Institute of Medical Sciences, Banaras Hindu University, Varanasi, Uttar Pradesh, India

**Keywords:** viral infection, corticosteroids, multiorgan failure, hyperinflammatory state, macrophage activation syndrome

## Abstract

Dengue virus is an arbovirus transmitted through the bite of infected Aedes mosquitoes. Many unusual clinical features are being reported in dengue. Dengue complicated with hemophagocytic lymphohistiocytosis (HLH) is a rare but potentially fatal condition. Here, we report an 18-year-old otherwise healthy female with dengue fever complicated with HLH. The diagnosis was made by fulfilling the clinical and laboratory criteria of HLH. She was managed successfully with a methylprednisolone pulse regimen. Our case highlights the importance of early recognition of complications and prompt treatment for a better outcome.

## Introduction

Dengue virus (DENV) is an arbovirus (ribonucleic acid virus), having five serotypes (DENV1, DENV2, DENV3, DENV4, and DENV5) in human which are transmitted through the bite of infected Aedes species mosquitoes. 1 2 Health care facilities are already overburdened due to the high prevalence of dengue in tropical and subtropical regions in Asia, the Pacific and Caribbean islands, and Central and South America. Many unusual, deadly complications such as intracranial hemorrhage have been reported in dengue. 2 Severe dengue is the major cause of death, resulting from hemorrhage, plasma leakage, fluid accumulation, respiratory distress, or organ dysfunction. 1 3 Dengue fever is associated with various atypical symptoms, including encephalitis, intracerebral infarction, facial palsy, intracranial hemorrhage, stomach hemorrhage, acute motor quadriparesis, and reversible blindness. 2 3 Additionally, hemophagocytic lymphohistiocytosis (HLH) in dengue is uncommon but is associated with increased mortality as high as 43%. 4

## Case Report

**Fig. 1:**
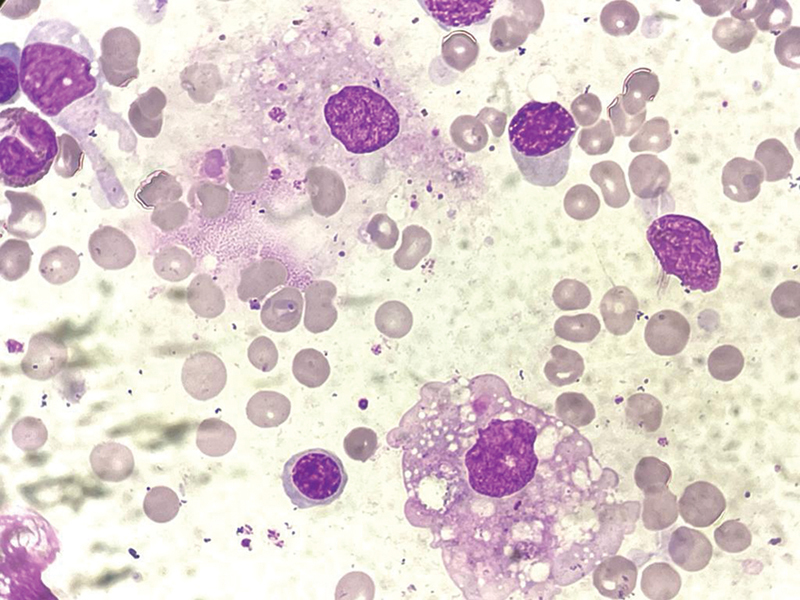
Bone marrow aspirates showing hypercellular marrow with erythroid hyperplasia and frequent hemophagocytic histiocytes.

An 18-year-old female was admitted with complaints of fever, body pain, headache, and weakness for 4 days and vomiting for 1 day. Clinically, she was hemodynamically stable. Her general and systemic examinations were unremarkable except for mild splenomegaly. There was no evidence of postural hypotension, a progressive rise in hematocrit, or the presence of extravasated fluid in the pleural or peritoneal cavity as determined by ultrasonography or clinical examination, therefore ruling out plasma leakage. Her initial investigations revealed mild anemia (11.5 g/dL; normal range [NR] 13–17), leucopenia (3.4 × 10 ^3^ /mm ^3^ ; NR 4–10), thrombocytopenia (0.73 × 10 ^3^ /mm ^3^ ; NR 15–410), elevated liver enzymes (alanine aminotransferase [ALT], 146 IU/L; NR < 45 and aspartate transaminase [AST], 781 U/L; NR 15–37), and normal prothrombin time (13.2 seconds) and activated partial thromboplastin time (32.3 seconds). Acute dengue fever was established by positive dengue nonstructural protein 1 (NS1) antigen. She had normal renal function, thyroid function, serum electrolyte, blood sugar, electrocardiography, chest X-ray, and urine analysis. Blood and urine cultures were sterile. Relevant clinical investigations ruled out other febrile causes. During hospitalization, she was managed with intravenous fluid and other symptomatic treatment. Despite conservative management, sustained high fever, decreasing cell counts (pancytopenia), and deteriorating liver function (ALT 546 IU/L and AST 1280 IU/L) provoke more suspected secondary HLH. On day 12 of illness (hospital day 8), bone marrow aspirate revealed frequent hemophagocytic histiocytes ( Fig. 1 ). Meeting criteria diagnosed HLH: fever (102.5°F), splenomegaly (mild), cytopenia (pancytopenia), hypertriglyceridemia (fasting triglyceride 627 mg/dL), and hyperferritinemia (ferritin 2650 ng/mL) and bone marrow findings of hemophagocytic histiocytes. We ruled out other causes of HLH. Intravenous dexamethasone (10 mg/m ^2^ /day) was added, but there was no improvement in clinical and laboratory parameters till 3 days. Dexamethasone was then replaced with intravenous methylprednisolone (MPS) pulse therapy (1 g) for 3 days, followed by oral prednisolone. She started to improve dramatically, and her fever subsided on day 2 of administering MPS (day 12 of hospitalization) and improved laboratory parameters. After that, she was discharged home in stable condition. Oral prednisolone was tapered to stop within 2 weeks.

## Discussion

HLH is an uncommon but potentially life-threatening syndrome characterized by hyperinflammation due to the uncontrolled proliferation of activated lymphocytes and histiocytes secreting large amounts of inflammatory cytokines. It can be divided into primary (genetic) or secondary (acquired). 5 The HLH is proposed due to the failure of cytolytic mechanisms by natural killer (NK) cells and cytotoxic T lymphocytes, which result in a hyperinflammatory state driven by the inability to clear the offending antigen and a consequent cytokine storm. 6

Primary HLH is associated with well-described genetic predispositions and is caused by gene mutations responsible for producing cytotoxic T cells and NK cells. These cells normally kill pathogen-infected cells. The primary form of HLH is often seen in children with genetic defects in NK and T cell cytotoxic activity. Genetic HLH refers to patients with a particular genetic anomaly, as shown in Table 1 . 7 8

**Table 1:** Genetic causes of HLH

Subtype	Gene/Protein	Location	Function
FHL1	Unknown	9q21.3-locus 6	Unknown
FHL2	*PFR1* /perforin 1	10q21–22	Cell lysis, membrane pore formation
FHL3	*UNC13D* /Munc 13–4	17q25	Cytolytic granule exocytosis
FHL4	*STX11* /syntaxin 11	6q24	Intracellular vesicle trafficking
FHL5	*STXB2* /syntaxin binding protein 2 or *UNC18B*	19p13	Intracellular vesicle trafficking
Griscelli syndrome type 2	*RAB27A* /Rab27a	15q21	Vesicle docking on microtubules
Chediak-Higashi syndrome	*LYST*	q42.1-q42.2	Vesicle maturation and sorting
Hermansky-Pudlak syndrome type 2	*AP3B1*	5q14.1	Encoding b subunit of AP3, vesicle maturation and transport
X-linked proliferative syndrome (XLP) type 1	*SHD2D1A* /SAP protein	Xp25	Polarization of cytolytic granules for transport to the immunological synapse
XLP type 2	*BIRC4* /XIAP protein	Xp25	Unclear

Abbreviation: HLH, hemophagocytic lymphohistiocytosis.

The secondary form occurs at older ages in individuals with concomitant conditions, including infection or cancer, without a genetic defect. The secondary HLH may be triggered by neoplastic, autoimmune, and infectious etiologies ( Table 2 ). 7 9 Infections are the most common cause of secondary HLH, and Epstein-Barr virus (EBV) is the most common infectious etiology. 9

**Table 2:** Acquired causes of HLH

Infectious etiologies	
Viral infections	EBV, CMV, human herpesvirus 8 (HHV-8), herpes simplex virus (HSV), varicella-zoster virus (VZV), human immune deficiency virus (HIV), human T-lymphotropic virus (HTLV), adenovirus, HAV, HBV, HCV, measles, mumps, rubella, dengue, hantavirus, parvovirus B19, parechovirus, enterovirus, H1N1 influenza virus, and recently severe acute respiratory syndrome coronavirus 2 (SARS-COV-2)
Bacterial infections	Staphylococcus aureus, Campylobacter spp., Fusobacterium spp., Mycoplasma spp., Chlamydia spp., Legionella spp., Salmonella typhi, Rickettsia spp., Brucella spp., Ehrlichia spp., Borrelia burgdorferi, Mycobacterium tuberculosis
Fungal infections	Candida spp., Cryptococcus spp., Pneumocystis spp., Histoplasma spp., Aspergillus spp., Fusarium spp.
Parasitic infections	Plasmodium falciparum, Plasmodium vivax, Toxoplasma spp., Babesia spp., Strongyloides spp., Leishmania spp.
Neoplastic disorders	
Hematological disorders	Peripheral T cell/NK-cell lymphomas, B cell lymphoma, anaplastic large-cell lymphoma (ALCL), acute lymphocytic leukemia, acute myeloid leukemia, Hodgkin lymphoma, multiple myeloma, acute erythroid leukemia, myelodysplastic syndrome
Nonhematological disorders	Prostate and lung cancer, hepatocellular carcinoma
Autoimmune disorders	Systemic-onset juvenile idiopathic arthritis, Kawasaki disease, systemic lupus erythematosus, seronegative spondyloarthropathies, Still's disease - juvenile and adult-onset, and rheumatoid arthritis
Iatrogenic conditions	Organ transplantation, chemotherapy, or immunosuppressive therapy

Abbreviations: CMV, cytomegalovirus; EBV, Epstein-Barr virus; HAV, hepatitis A virus; HBV, hepatitis B virus; HCV, hepatitis C virus; HLH, hemophagocytic lymphohistiocytosis; NK, natural killer.

After the genetic form, Risdall et al first characterized acquired HLH in 1979 in individuals with viral infections after organ donation. 10 Later, it was shown that HLH can also affect immunocompetent people. Previously thought to occur in adults, acquired HLH can affect children of any age. 7

Another entity called macrophage activation syndrome (MAS) was first linked to a pediatric rheumatic disease in 1985 but may have been first described as early as the mid-1970s. 11 MAS refers to HLH that results from autoimmune disorders. Patients may show HLH-like symptoms, although severe coagulopathy and cardiac dysfunction are common presentations. The pathophysiology of MAS is likely impaired NK/T cell function, similar to other kinds of HLH. 7

For establishing HLH, at least five criteria should be fulfilled out of the eight listed below. These include (1) fever, (2) splenomegaly, (3) cytopenia affecting at least 2 of 3 lineages in peripheral blood, (4) ferritin ≥ 500 μg/L, (5) hypertriglyceridemia and/or hypofibrinogenemia, (6) hemophagocytosis in bone marrow or spleen or lymph nodes, (7) low or absent NK-cell activity, and (8) high level of soluble CD25. A highly elevated serum ferritin level is strongly related to HLH with a cutoff value of > 10,000 mcg/L having 90% sensitivity and 96% specificity. Whereas hyperferritinemia in dengue infection indicates a highly active disease with an increased risk of hyperinflammation and coagulation disturbances. 5

Dengue fever with atypical and unusual manifestations, not classified as dengue shock syndrome or dengue hemorrhagic fever, is known as expanded dengue syndrome. 1 2 HLH is an uncommon hematological symptom of expanded dengue illness, alongside disseminated intravascular coagulopathy and cytopenias. 12

Our patient had features of dengue, including fever, acute frontal headache, discomfort, arthralgia, and myalgia. NS1 antigen positive verified the diagnosis, enabling continued treatment. Fever persisting after dengue infection may result from sepsis and expanded dengue syndrome, including HLH. 12 Further, the workup ruled out sepsis, and the patient's bone marrow showed considerable hemophagocytic activity. The patient met the criteria confirming HLH diagnosis. The proposed treatment was initiated shortly.

HLH-94 protocol is for acutely ill or clinically deteriorating patients. For clinically stable patients, initial empiric treatment for the suspected underlying condition, rather than the HLH-94 protocol should be considered, examples include antimicrobial agents for triggering infections, glucocorticoids for rheumatologic disorders, or antineoplastics for cancers. The management of HLH involves suppressing inflammation, eliminating immune cells, eliminating triggers, supportive therapy (neutropenia, coagulopathy), and replacing the dysfunctional immune system. The 2004 Histiocyte Society's second international meeting protocol suggests an 8-week induction therapy with corticosteroids, etoposide, and cyclosporine A for HLH treatment. 13

The first choice for suppressing hypercytokinemia is corticosteroids. Dexamethasone, the first-line treatment, performs better than prednisolone in suppressing central nervous system inflammation due to its more blood–brain barrier crossing. 14

The trigger can be eliminated with anti-infectious therapy: a methotrexate upfront and intrathecal therapy for selected patients. Further hematopoietic stem cell transplantation is advised for individuals with familial disease, established molecular diagnosis, or severe, chronic, or reactivated disease. 13

In dengue-associated HLH, pulse dosages of MPS or dexamethasone are typically administered to reduce hyperinflammatory symptoms. However, some patients have recovered spontaneously with supportive care. Treatment of dengue-induced HLH with intravenous immunoglobulin G shows favorable outcomes. 15

Without treatment, hereditary HLH has a devastating prognosis, with a median survival of 1 to 2 months and a likelihood of less than 10% for 3 years. 7 16 EBV has the poorest prognosis among viruses linked to HLH, with reported fatality rates ranging from 25 to 100%. 7 However, adding etoposide to the therapeutic regimen has shown positive outcomes, particularly if started during the first 4 weeks. 17

## Conclusion

HLH is an uncommon syndrome in dengue but a potentially life-threatening condition. Therefore, the treating clinician must be aware and have a high suspicion of HLH in patients not responding to standard supportive therapy and with persistent fever and cytopenias. Early recognition and appropriate management with corticosteroids contribute to the successful outcome of dengue fever complicated by HLH.
